# Fast forward genetics to identify mutations causing a high light tolerant phenotype in *Chlamydomonas reinhardtii* by whole-genome-sequencing

**DOI:** 10.1186/s12864-015-1232-y

**Published:** 2015-02-06

**Authors:** Lisa Schierenbeck, David Ries, Kristin Rogge, Sabrina Grewe, Bernd Weisshaar, Olaf Kruse

**Affiliations:** Department of Biology/Center for Biotechnology, Algae Biotechnology and Bioenergy, Bielefeld University, Universitätsstrasse 27, 33615 Bielefeld, Germany; Department of Biology/Center for Biotechnology, Genome Research, Bielefeld University, Universitätsstrasse 27, 33615 Bielefeld, Germany

**Keywords:** Whole-genome-sequencing, *Chlamydomonas reinhardtii*, Forward genetics, Mutation identification, SNPs, High light

## Abstract

**Background:**

High light tolerance of microalgae is a desired phenotype for efficient cultivation in large scale production systems under fluctuating outdoor conditions. Outdoor cultivation requires the use of either wild-type or non-GMO derived mutant strains due to safety concerns. The identification and molecular characterization of such mutants derived from untagged forward genetics approaches was limited previously by the tedious and time-consuming methods involving techniques such as classical meiotic mapping. The combination of mapping with next generation sequencing technologies offers alternative strategies to identify genes involved in high light adaptation in untagged mutants.

**Results:**

We used the model alga *Chlamydomonas reinhardtii* in a non-GMO mutation strategy without any preceding crossing step or pooled progeny to identify genes involved in the regulatory processes of high light adaptation. To generate high light tolerant mutants, wildtype cells were mutagenized only to a low extent, followed by a stringent selection. We performed whole-genome sequencing of two independent mutants *hit1* and *hit2* and the parental wildtype. The availability of a reference genome sequence and the removal of shared bakground variants between the wildtype strain and each mutant, enabled us to identify two single nucleotide polymorphisms within the same gene Cre02.g085050, hereafter called *LRS1 (putative Light Response Signaling protein 1)*. These two independent single amino acid exchanges are both located in the putative WD40 propeller domain of the corresponding protein LRS1. Both mutants exhibited an increased rate of non-photochemical-quenching (NPQ) and an improved resistance against chemically induced reactive oxygen species. *In silico* analyses revealed homology of LRS1 to the photoregulatory protein COP1 in plants.

**Conclusions:**

In this work we identified the nuclear encoded gene *LRS1* as an essential factor for high light adaptation in *C. reinhardtii*. The causative random mutation within this gene was identified by a rapid and efficient method, avoiding any preceding crossing step, meiotic mapping, or pooled progeny. Our results open up new insights into mechanisms of high light adaptation in microalgae and at the same time provide a simplified strategy for non-GMO forward genetics, a crucial precondition that could result in the identification of key factors for economically relevant biological processes within algae.

## Background

The unicellular eukaryotic green alga *C. reinhardtii* is a well-studied model organism and has also great potential for the utilization in biotechnological applications (for details see: [1-4]). Phototrophic large scale production of microalgae biomass for biotechnological purposes under outdoor conditions depends on the development of new highly robust algae species tolerating abiotic stresses including higher light intensities. A broad molecular toolkit, predominant haploid life cycle, and a fully sequenced genome [5,6] opens various opportunities for genetic engineering to obtain optimized strains of *C. reinhardtii*. For many biotechnological applications it is, however, preferred to use optimized, but not genetically modified organisms (GMO) in order to avoid restrictions for outdoor cultivation [7]. Of particular interest is the identification of more robust non-GMO strains showing certain tolerance against variations in temperature and light intensity [8]. Over recent years several efforts have been reported to identify and construct algae strains with improved light conversion efficiency rates (PCE rates) [9-16] as well as more robust variants surviving elevated and fluctuating light conditions, which is crucial for establishing efficient outdoor cultivation [8,17-19]. Such fluctuating or high light regimes cause an imbalance in the absorbtion and utilization of light energy that can lead to photooxydative damage due to the production of reactive oxygen species (ROS) resulting in severe cell damage or even cell death [18,20-23] The rather complex regulation of light adaptation mechanisms is not yet fully understood and key factors still need to be identified.

Forward genetic approaches with non-GMO strains, however, have been limited so far by the tedious and time-consuming methods of classical meiotic mapping to identify the underlying genotype. With the advent of next generation sequencing technologies, the identification of causative mutations has been greatly facilitated. Currently, most recent advanced approaches are essentially based on three different strategies [24-29]: either (i) meiotic mapping by bulked segregant analysis is combined with whole genome sequencing in a one-step approach to narrow down the causative genomic locus [30-33]; (ii) unlinked mutations are removed by backcrossing to the wildtype (WT) stain prior to sequencing [34-38] or (iii) mutations are identified by direct sequencing of two or more allelic mutants [39].

The development of the first strategy, enabling fast forward genetics in a mapping-by-sequencing approach, was first published by Schneeberger *et al*. [30] (SHOREmap)*.* They outcrossed *Arabidopsis thaliana* mutant strains to a well-characterized polymorphic WT strain and sequenced a pool of 500 F_2_ progeny carrying the mutant phenotype. Regions unlinked to the mutation underlying the phenotype are heterozygous due to meiotic recombination, whereas in regions linked to the mutation, the marker SNP (single nucleotide polymorphism) frequency is biased towards the mutant variants. By plotting the relative allele frequencies of the two mapping parents on the pseudochromosomes, they were able to narrow down the region of interest and identify the causative mutation. A similar approach was applied in *Caenorhabditis elegans* [31], demonstrating that sequencing of a pool of only 20 progeny carrying the phenotype is sufficient to identify the causative mutation. Quite recently, this method was adapted even to the large genomes of some vertebrates such as zebrafish and mouse [24,33,36]. The effort of meiotic mapping was thereby greatly reduced, however, the strategy still requires crossing to a polymorphic strain and sequencing of pooled progeny.

A more straightforward and faster approach would be the direct comparison of mutant and WT genomes or of two or more allelic mutants [26,28,39]. In order to obtain isogenic strains, the mutants are backcrossed to the starting strain used for mutagenesis prior to sequencing to remove any unlinked mutations. Re-sequencing and subsequent subtraction of common variants from the datasets of single mutants and/or WT with a similar genetic background was proven to be sufficient to identify the causative mutation for example in fission yeast [40], *Drosophila* [34], *C. elegans* [35] and *Arabidopsis* [38]. Moreover, mutagen-induced SNPs themselves can be used as new mapping markers [34,35,38]. The advantage of this method is clearly that no polymorphic strain or pooled progeny is needed, because, in this case, single mutant and WT strains are simultaneously sequenced. To circumvent the need for a reference genome, Nordström *et al*. [39] introduced the algorithm NIKS (needle in the *k*-stack), allowing identification of mutations even in the absence of a reference sequence after backcrossing to the WT and sequencing of pooled F_2_ progeny.

All these strategies, however, require out- or backcrossing of the mutant strains either to a highly polymorphic strain for meiotic mapping or to the parental WT strain to remove unlinked mutations. It is obvious that if the crossing step could be omitted, the overall time and effort needed for mutant identification would be considerably reduced. Nordström *et al*. [39] therefore also applied NIKS to compare the genomes of two allelic mutants that were isolated in the same screen using ethyl methanesulfonate as mutagen and form a complementation group with all F_1_ progeny showing the mutant phenotype. They sequenced 35 pooled M_3_ plants, derived from seeds after after self-pollination of each original mutant plant. By searching for genes carrying mutations in both mutants they were able to unambiguously identify the causal gene.

In our approach, we intended to identify new genes involved in the regulation of high light adaptation. For this purpose, we sequenced two high light (HL) resistant *C. reinhardtii* mutants that originated from a forward genetic approach by either spontaneous mutation or ultraviolet (UV) light induced mutagenesis, respectively. The mutants were selected in a condition lethal to WT cells, with only few mutants surviving the screening. By applying this strategy, the mutational load was considered to be rather low. We performed whole-genome sequencing of the closely related mutant strains and the parental WT. The availability of a reference genome sequence and subsequent subtraction of common variants between each mutant and the WT enabled us to identify a single nucleotide mutation in the predicted gene Cre02.g085050 in both mutants. The encoded protein LRS1 is presumably involved in the regulation of (high) light response in *C. reinhardtii*. We hereby successfully identified a potential key factor essential for functional light adaptation by applying a simplified strategy for the fast identification of single mutations to a microalgal species. The identification of *LRS1* will provide new insights into mechanisms essential for high light tolerance. Furthermore, our approach may serve as an example for the fast identification of genes of interest in untagged mutants with phenotypes that can be selected for, a crucial precondition that could finally yield in the identification of genetic factors responsible for the underlying biological processes of desirable phenotypes.

## Results

### Generation and phenotypical characterization of high light tolerant *C. reinhardtii* mutants

**Figure 1:**
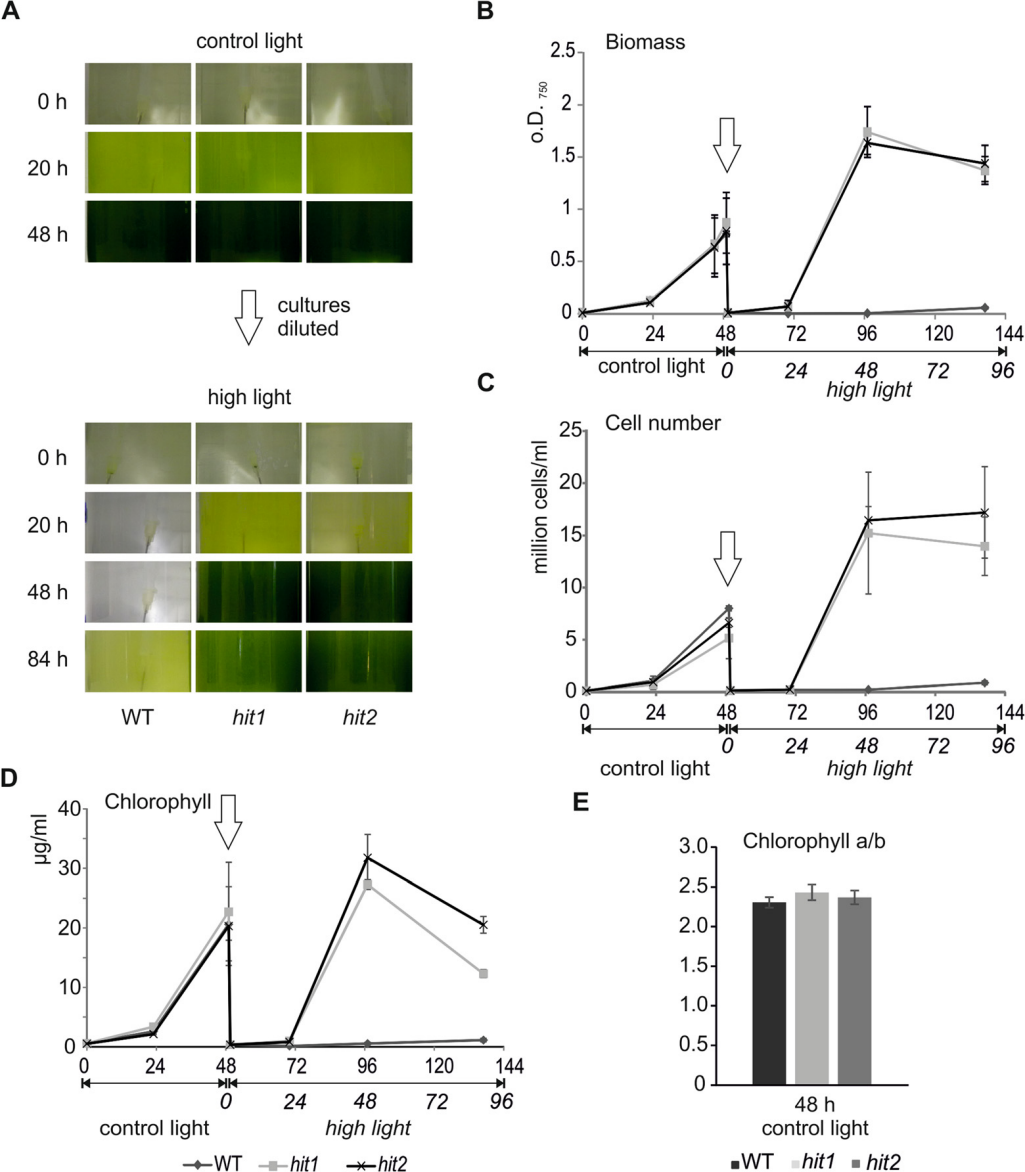
**Photoautotrophic growth of two high light tolerant mutants** ***hit1*** **and** ***hit2*** **and the parental WT.** Growth was monitored under control light (600 μmol m^-2^ s^-1^) and HL conditions (2500 μmol m^-2^ s^-1^) as **(A)** photographic images of the cultures during the experiment, **(B)** the biomass of the cultures (determined by optical density of the culture at 750 nm), **(C)** the cell number per ml culture and **(D)** the amount of chloropyll per ml culture with **(E)** the chlorophyll a/b ratio at control light. The arrow indicates the dilution of the cultures and the switch to HL conditions.

Tolerance to fluctuating light conditions, in particular to high light stress, is a desired phenotype for microalgae that are intended to be used for outdoor cultivation in biotechnological approaches [8]. In order to obtain non-GMO high light tolerant *Chlamydomonas* strains, we applied a selection method under light intensities of 1500–2000 μmol m^-2^ s^-1^ that are known to be lethal to WT cells (see: [41]). For mutant generation the cells were either treated with UV light and then exposed to HL, or directly subjected to HL without any pre-treatment (spontaneous mutants). Most cells did not survive the HL conditions (Table 1), but a limited number of single green colonies appeared and were isolated two weeks after the onset of the selection. On control plates without any selection no limitation in cell growth could be observed. In a subsequent screening, the cells were cultivated under photoautotrophic conditions with very high light intensities of 2500 μmol m^-2^ s^-1^. While the WT cells did not survive these conditions or at least showed a very prolonged lag phase, the selected mutants *hit1* and *hit2* (*high light tolerant*) exhibited a robust growth phenotype, with growth rates similar to the WT under light conditions optimal for photoautotrophic growth (600 μmol m^-2^ s^-1^ [41]) (Figure 1).

**Table 1:** **Selection and screening of high light tolerant** ***C. reinhardtii*** **cells**

**UV light treatment**	**Cells before treatment**	**Colonies after high light selection**	**Colonies screened**	**High light tolerant**
no	4 000 000	73	4	3
3 min	4 000 000	2	2	1
5 min	4 000 000	115	5	5
10 min	4 000 000	6	4	3
15 min	4 000 000	9	6	4

**Figure 2:**
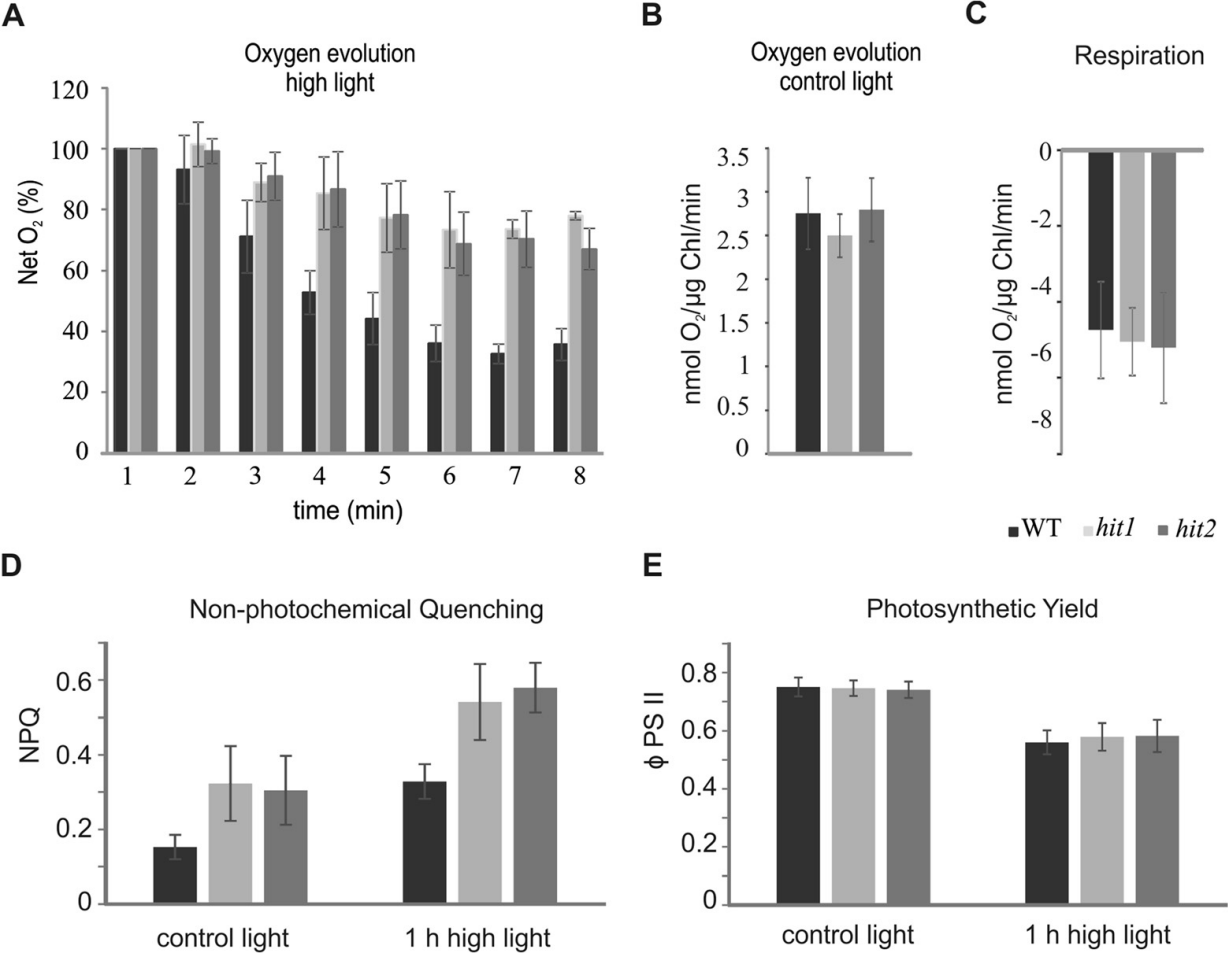
**Photosynthetic activity of the mutants compared to the WT. (A)** Decline of the net oxygen evolution of cultures adapted to control light (400 μmol m^-2^ s^-1^), when exposed to high light (2500 μmol m^-2^ s^-1^) during the measurement. **(B)** Net oxygen evolution rate at control light and **(C)** average respiration rate in the dark before and after the high light treatment **(D)** Non photochemical quenching and **(E)** photosynthetic yield under control light and after one hour of high light treatment.

**Figure 3:**
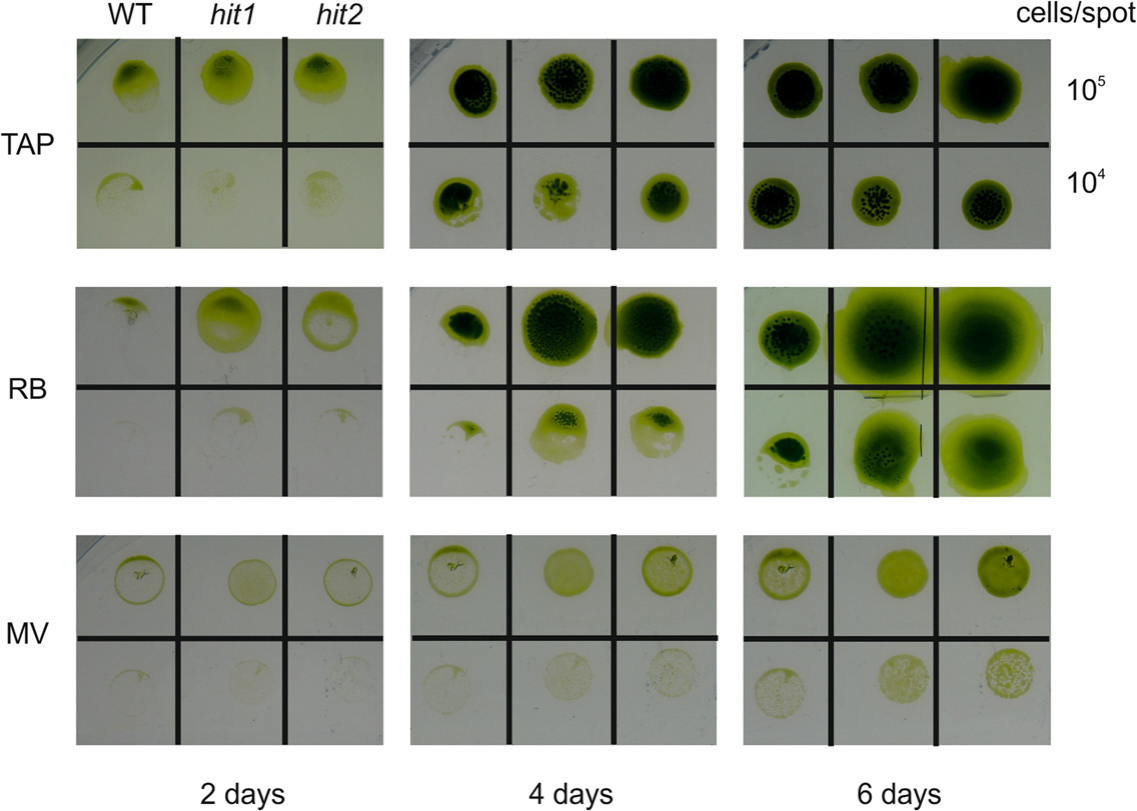
**Sensitivity of the mutants** ***hit1*** **and** ***hit2*** **and the WT against reactive oxygen species.** Growth of the cells after 2, 4 and 6 days on control plates with TAP medium and on plates containing the two reactive oxygen species singlet oxygen, induced by rose bengal (RB), and superoxide anion radicals induced by methyl viologen (MV).

To investigate the phenotype of these mutants, we measured the *in vivo* oxygen evolution activity with a Clark-type O_2_ electrode. When monitoring the activity of low light adapted cells under very HL conditions, we observed a rapid reduction of net oxygen evolution during the measurement (Figure 2A), with no altered oxygen evolution rate in control light (Figure 2B) and comparable respiration rates (Figure 2C). The decline of photosynthetic activity in high light was slower and less pronounced in the mutants, underlining their robust phenotype under HL when compared to the WT. While the WT exhibited a 53% reduction after 4 min onset of HL, the mutants retained approximately 70% of their photosynthetic activity even after 8 min of HL treatment. Additionally, the mutants exhibited an increased rate of non-photochemical-quenching (NPQ) compared to the WT during growth under both conditions, control light and 1h HL treatment (Figure 2D) but a similar photosynthetic yield (Figure 2E). Moreover, the mutants showed an improved resistance against chemically induced ROS, especially singlet oxygen, when compared to the WT (Figure 3).

The two HL tolerant mutants *hit1* and *hit2*, as well as the corresponding WT strain, were eventually investigated in a whole-genome re-sequencing approach to systematically identify the genetic background of the HL tolerant phenotype.

### Genotypical characterization of *hit1* and *hit2* by whole genome re-sequencing

**Figure 4:**
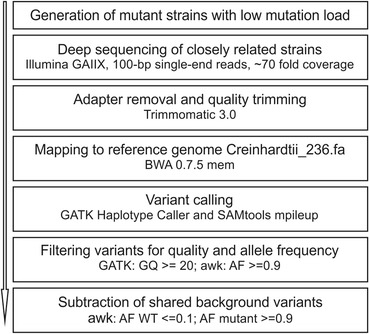
**Schematic representation of the workflow applied for mutation analysis.** BWA: Burrows-Wheeler Aligner; GATK: Genome Analysis Toolkit; GQ: Genome Quality; AF: Allele frequency.

For the systematic identification of the mutation(s) responsible for the HL tolerant phenotype in *hit1* and *hit2*, we applied a methodical workflow (Figure 4) based on open source bioinformatics tools that allow quality filtering as well as sorting out common variants. Sequencing of the parental WT strain and the closely related mutant side-by-side enabled us to remove all variants in each mutant that are also present in the background strain. Whole-genome sequencing was performed on an *Illumina Genome Analyzer IIx* to obtain a theoretical 70-fold coverage for the mutants and 110-fold coverage for the parental WT reference strain CC124 (mating type minus, mt-). In contrast to most previously presented strategies, the strains were not crossed or pooled prior to sequencing. All sequences were trimmed and mapped to the C*hlamydomonas* assembly v5 of the DOE Joint Genome Institute (JGI) (reference strain CC503, mt+). After trimming and quality filtering the obtained coverage was 84.18 for the WT and 60.93 and 58.99 for the mutants *hit1* and *hit2*, respectively, with ~85% covered by at least 15 reads (Table 2). SNPs (including small indels) were called using the GATK Haplotype Caller as well as SAMtools mpileup. For quality filtering we used the Genotype Quality filter (GQ), which estimates the probability that the genotype of each sample is correct. Ness *et al.* [42] thoroughly tested various quality filters and settings for *C. reinhardtii* and concluded that GQ of 20 or greater was the optimal threshold for minimizing both false negatives and positives. Moreover they excluded all heterozygous sites, as *C. reinhardtii* is haploid and heterozygous sites likely represent alignment or sequencing errors. After applying the quality cutoff GQ ≤ 20 and removal of heterozygous sites, we obtained 93,585 variants. In a next step, common SNPs between each individual mutant and the parental WT strain were deleted from this dataset. With this final filtering process and due to the low load of mutations, we detected only four variants (three substitutions and one insertion) that differed between the strains (Table 3).

**Table 2:** **Sequence coverage of WT,** ***hit1*** **and** ***hit2*** **obtained by using the GATK** ***DepthOfCoverage*** **profiler**

**Sample**	**Total number of mapped bases**	**Mean fold coverage**	**Covered by ≥ 15 bases (%)**
WT	9 011 680 500	84.18	87.6
*hit1*	6 522 916 358	60.93	85.1
*hit2*	6 314 509 607	58.99	84.4

**Table 3:** **Identified variants that are specific for the respective mutant (Chr: Chromosome; nt: nucleotides; CDS: Coding Sequence)**

**Mutant**	**Variant position**	**Type**	**CDS**	**Affected gene**
*hit1*	Chr2: 1596893	Substitution (1nt)	Yes	Cre02.g085050
*hit2*	Chr2: 1597931	Substitution (1nt)	Yes	Cre02.g085050
*hit1*	Chr8: 2390449	Insertion (2nt)	Intron	Cre08.g371000
*hit1*	Chr12: 2939906	Substitution (1nt)	Yes	Cre12.g501600

We identified only one mutation in *hit2* compared to the WT, and the affected gene Cre02.g085050 (*LRS1*) is consequently the best candidate to cause the high light tolerant phenotype in *hit2*. This finding was very much supported by the determination that the same gene was also mutated in the mutant *hit1*. In this mutant two substitutions and one insertion were identified. Apart from the substitution in LRS1, we identified another substitution on chromosome 12 (Cre12.g501600) and an insertion of two nucleotides on chromosome 8 (intronic region of Cre08.g371000). We cannot fully rule out the possibility that the two other identified mutations contribute to the phenotype in *hit1*, but the fact that two independent mutants with the same phenotype show lesions in the same gene strongly indicates that the identified mutations in LRS1 are causative for the phenotype in both mutants. Furthermore, the substitution on chromosome 12 is causing a silent mutation and the insertion on chromosome 8 is located in a highly repetitive region, as detected with the software RepeatMasker.

**Figure 5:**
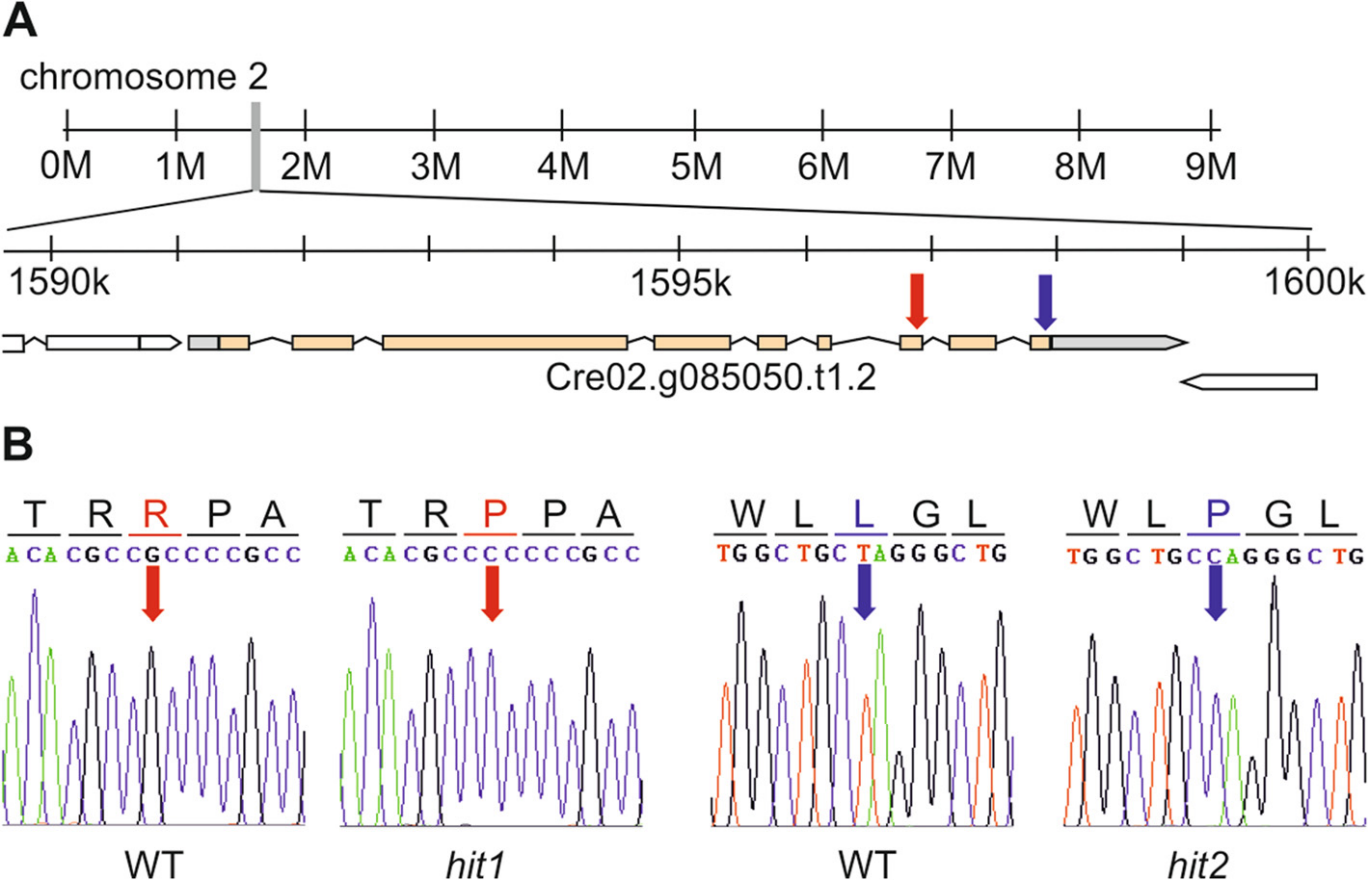
**Localization and confirmation of the identified mutations. (A)** Schematic genomic view of chromosome 2 (1591109–1599021) derived from Phytozome version 9.1 [94] with arrows indicating the localization of the mutation in *hit1* (1596839, red arrow) and *hit2* (1597931 blue arrow) within the Cre02.g085050 transcript. **(B)** Confirmation of the mutation by sanger-sequencing in *hit1* and *hit2* compared to WT (arrows pointing the nucleotide exchange) with the corresponding amino acids.

In both mutants the gene *LRS1* (Cre02.g085050) on chromosome 2 (Figure 5A) was affected by a single nucleotide substitution in the exonic regions of a predicted WD40 domain. In *hit1* we identified a nucleotide exchange from guanine to cytosine that leads to a replacement of the arginine residue R_1256_ with proline. The identified mutation in *hit2* is a nucleotide substitution from thymine to cytosine corresponding with an amino acid substitution from leucine L_1439_ to proline. Both SNPs were confirmed by Sanger-sequencing (Figure 5B).

We furthermore surveyed if sequencing of only the mutants without the WT would have been already sufficient to identify the specific variants in each mutant. Therefore, we compared the SNPs of the two mutants directly to each other. This data processing resulted in the identical SNPs as determined by comparing the WT with one of the mutants. We consequently concluded, in accordance with the results of Zuryn *et al*. [35] that sequencing of the parental WT is not even required to identify the mutations if two closely related mutant strains with the same background are sequenced in parallel.

### Confirmation of the causative mutation in *LRS1* referring to HL tolerance

**Figure 6:**
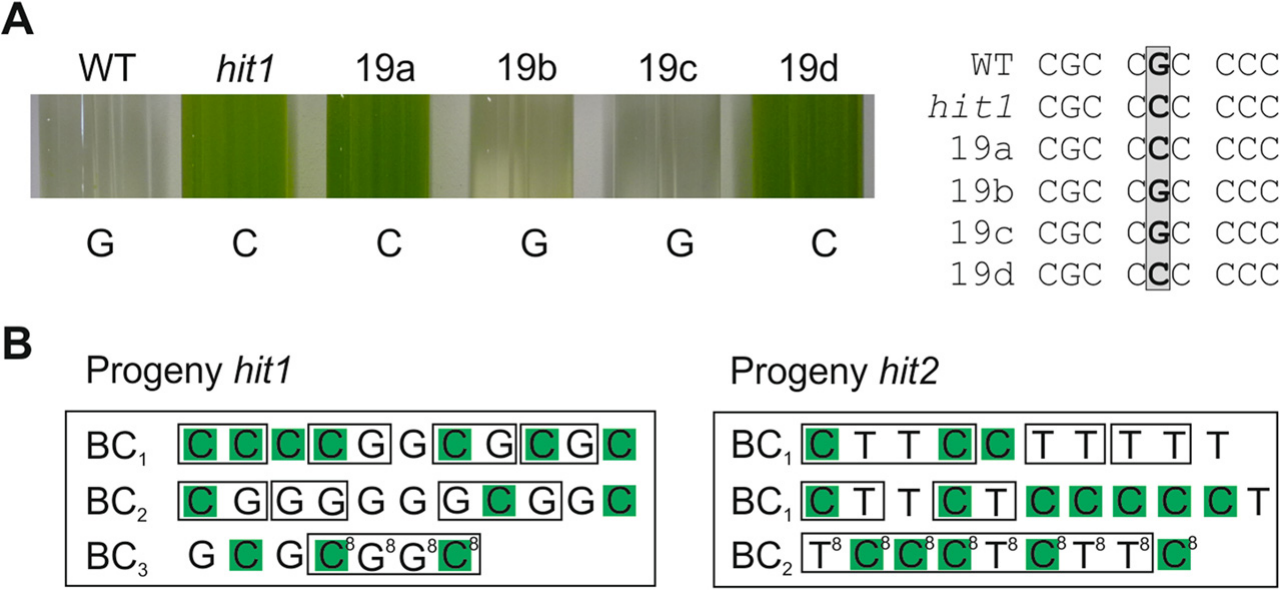
**Segregation of the mutant phenotype and the corresponding SNP in backcrossed mutants. (A)** Phenotype after 24 h in HL of WT, *hit1* and 4 representative progeny of the third round of backcrossing (19a-d, obtained from an incomplete octad) and an alignment of the corresponding DNA sequences obtained by Sanger-sequencing with the SNP highlighted in grey. **(B)** Sanger sequencing results for all tested progeny showing the respective nucleotide at position 1596839 (G in WT, C in *hit1*) and 1597931 (T in WT, C in *hit2*) on chromosome 2 and HL-tolerant phenotype highlighted in green. BC_1–3_ refers to backcrosses 1 to 3, small boxes in (B) indicate progeny that were derived from one zygote, octads are marked with (^8^).

To further verify that the identified mutations within the gene *LRS1* are indeed linked to the HL tolerant phenotype, we backcrossed the mutants to the WT strain CC125 (mt +). HL tolerant progeny were crossed again to the WT CC124 (mt-) or CC125 (mt+) depending on the mating type, which is not linked to the phenotype. Most of the meiotic tetrads or octads were incomplete; we obtained only one complete tetrad from the first crossing of *hit2* and one complete octad from the second crossing of *hit2*. This quite low yield of complete tetrads did not impair our analysis since we did not perform the crossing in order to identify the mutations by tetrad analysis, rather only to check for the segregation of the mutation with the phenotype. Nevertheless, in the progeny obtained from the complete tetrad or octad, a clear 2:2 pattern was observed as one could expect from a single, nuclear mutation causing the phenotype (Figure 6B). In total, 29 and 30 progeny from up to three rounds of backcrossing of *hit1* and *hit2* were checked for their phenotype, respectively. DNA of all the progeny and the corresponding parental strains (*hit1*, *hit2*, CC124, and CC125) was amplified within the identified SNP-containing region of each mutant and the resulting PCR products were Sanger-sequenced. As a result, the corresponding nucleotide exchange could be confirmed in all HL tolerant progeny and mutant strains, whereas, in contrast, there was no exchange in the progeny that exhibited a HL sensitive phenotype (Figure 6). Based on these results, with no other non-synonymous SNPs identified in the mutant *hit1*, plus the fact that two analysed mutants with the same phenotype have lesions in the same gene, we obtained sufficient evidence that the nucleotide changes in *LRS1* are responsible for the observed HL tolerant phenotype in both mutants. The observed single mutations in *hit1* and *hit2* within LRS1 could have caused both, gain or loss of function. Complementation experiments and the generation of vegetative diploids can therefore be considered as being part of future functional analyses regarding the regulatory role of LRS1 in high light adaptation.

### Localization of the mutations in a predicted WD40 ß-propeller motif of LRS1

**Figure 7:**
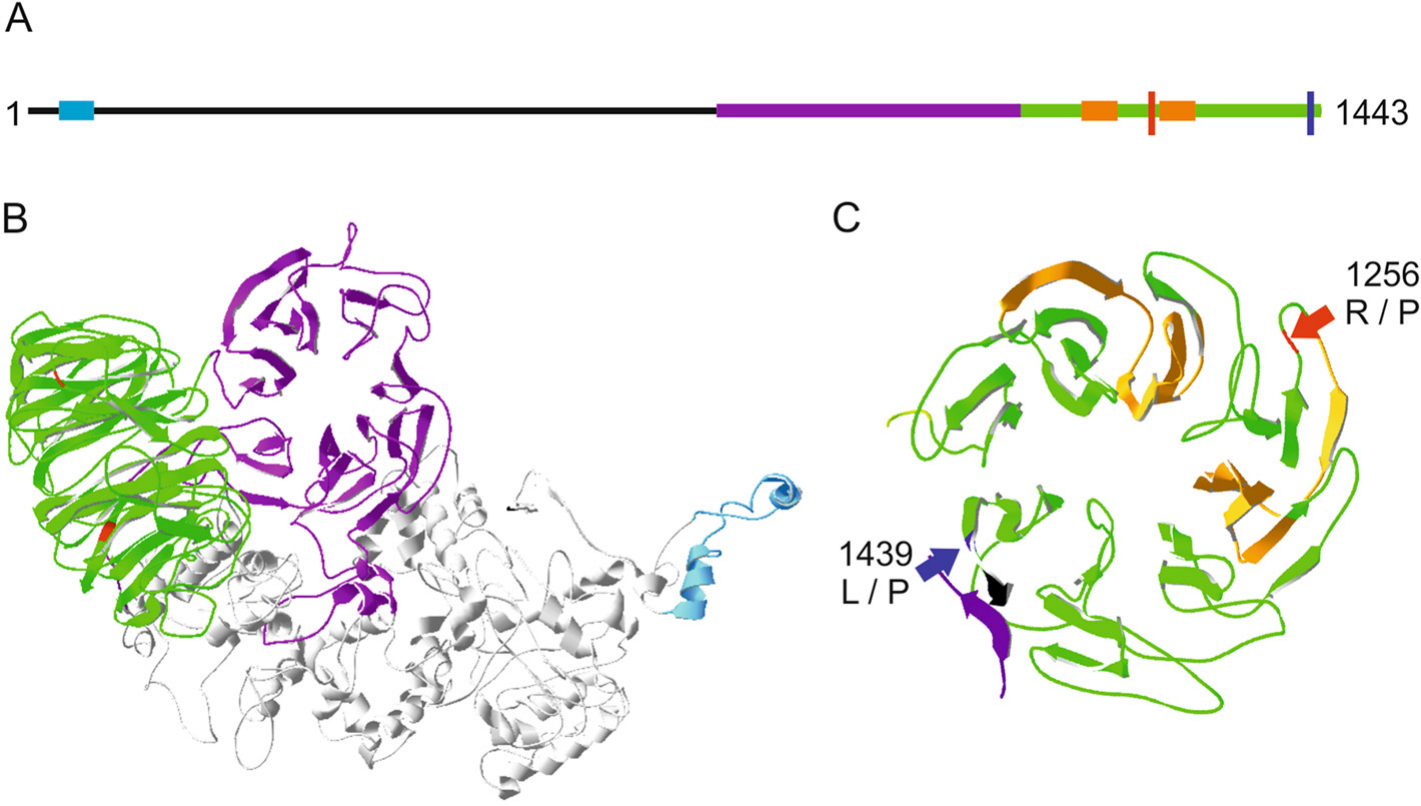
***In silico*** **analysis of the protein structure of LRS1. (A)** Position of the annotated *Pfam* domains with the C_3_HC_4_ type zinc finger (RING) domain (Pfam:00097) at the N-terminus (blue), two repeats of a WD40 domain (Pfam:00400) at the C-terminal end (orange), the position of the two ß-propeller domains predicted by the model as shown in B (purple and green) and the position of the mutation in *hit1* (red) and *hit2* (blue). **(B)** *Ab initio* model based on the amino acid sequence of the protein generated with I-TASSER. The RING domain is colored in blue, the seven-fold propeller in purple and the eight-fold propeller harboring the mutations in green, using the DeepView-Swiss-PdbViewer. **(C)** Top view of the eight-fold propeller structure with the localization of the annotated WD40 repeats (orange) and the two mutations identified in *hit1* (red arrow) and *hit2* (blue arrow).

According to the *Phytozome* annotation the predicted *LRS1* protein consists of 1443 amino acids and contains two known domains: a C_3_HC_4_ type zinc finger (RING) domain (Pfam:00097) at the N-terminus and two repeats of a putative WD40 domain (Pfam:00400) at the C-terminal end (Figure 7A). WD40 domains form a ß-propeller that normally consist of seven to eight propeller folds (each a repeat of ~40 amino acids) [43,44]. RT-PCR performed with primers deriving from this ß-propeller domain region confirmed transcription of *LRS1* in all strains (data not shown). In accordance to this data, recent RNA-seq data also showed the existence of *LRS1* mRNA in *C. reinhardtii* and revealed an upregulation of the transcript level after a shift from dark to light [45].

To gain more insights into the mutation sites of LRS1 causing the HL tolerant phenotype, a three dimensional *ab initio* model based on the amino acid sequence of the protein was generated with I-TASSER (Figure 7B and C). The predicted accuracy of the model is in a good range with a confidence score of −0.83 and a TM-Score of 0.61. The two WD40 repeat motifs that were already predicted through similarities of the amino acid sequence itself are represented in the model as two folds of an overall eight-fold ß-propeller at the C-terminus of the protein. In addition to this eight-fold propeller, a second putative seven-fold ß-propeller, not predicted before by the amino acid sequence, was predicted by the model. Of particular interest for this work was, however, that the identified point mutations in the mutants *hit1* and *hit2* are located within the same motif, both causing amino acid substitutions in the putative WD40 domain (Figure 7C).

It should be noted that a similar arrangement of the RING domain at the N-terminus and a WD40 propeller at the C-terminus can be found in the photoregulatory protein COP1 in other organisms [46]. A sequence alignment of proteins similar to COP1 of different phototrophic organisms and LRS1 reveals high sequence similarity in conserved regions of the functional domains, intermitted by additional regions in LRS1 with no sequence similarity. In higher plants, the COP1 protein is known to be a key regulator of the light signaling pathway [47] thus providing first hints towards a potential involvement of LRS1 in cellular light adaptation processes, which is fitting with the observed phenotype.

## Discussion

### Whole genome sequencing to identify a gene involved in high light adaptation in *C. reinhardtii*

Forward genetic approaches depend on the rapid detection of the underlying genotype. Techniques to identify mutations induced by insertional mutagenesis are very sophisticated [48,49]. On the contrary, strategies that intend to avoid foreign DNA as mutagen by randomly creating new phenotypes (and also allelic series with the option to mutagenize essential genes of interest) are hindered by the laborious and time consuming methods of meiotic mapping in order to identify the disrupted gene in a selected strain. Next generation sequencing provides a powerful technology to identify mutations such as SNPs and small indels as already demonstrated in various model organisms over the past few years [28-31,33-40,50]. The strategy of combined meiotic mapping and whole genome sequencing exceedingly reduced the effort to identify causative mutations [30]. Nevertheless, strains must be amenable to crossing and a polymorphic strain is also required. Moreover, some phenotypes are sensitive to the genetic background [26,38,51] and pooling of several mutants may not always be feasible [35]. To circumvent the need of a characterized polymorphic strain, Zuryn *et al*. [35] presented another strategy for *C. elegans*. They backcrossed their mutant strains to the original non-mutagenized strain 4 to 6 times, and directly sequenced three mutants side-by-side. Common nucleotide variants that were shared between at least two of the three mutants were subsequently subtracted. They concluded from their results that less sequencing coverage and fewer backcrosses may suffice, but this was not further tested in this study. Nordström *et al*. [39] furthermore demonstrated that mutation identification is feasible even without relying on any kind of recombination by searching for common genes that are disrupted in two different mutants with the same phenotype derived from the same mutagenesis screen.

As a further step towards a fast and straightforward identification of causative mutations, we now show that, in the microalgal model system *C. reinhardtii*, direct sequencing of only two mutants and the closely related WT side-by-side allows the identification of a causative mutation. In our approach, the number of the identified SNPs was successfully reduced by subtraction of background nucleotide variants that are shared between the WT and a mutant strain. This was possible due to a considerably low mutational load resulting from spontaneous mutation or low intensity UV mutagenesis followed by a very restrictive selection. By omitting the preceding crossing step and sequencing single mutants, the overall time needed for the identification of the mutation could be greatly reduced. We furthermore demonstrated that sequencing of the parental WT is not even required to identify the mutations if two closely related mutant strains with the same background are sequenced in parallel.

To achieve such a low amount of variants that differ between the strains as observed in our study, the strains need to be closely related, for example two different mutants that are derived from the same starting strain and mutagenesis screen [35,38,39]. If no closely related strain is available and the mutant strains are compared only to the reference sequence, the number of SNPs will remain very high. Even in the comparably small and haploid *Chlamydomonas* genome, simple comparison of the mutants with only the reference genome is not sufficient to identify the causative mutations [52,53] due to the genetic variation between different *Chlamydomonas* WT strains [54]. In this case the identification of causative mutations is still feasible, however with the disadvantage that several strains need to be sequenced to remove a sufficient number of common SNPs. With more sequencing data available for an ever increasing number of strains, large SNP libraries will likely obviate the need of closely related strains in future approaches [36,53].

From our data, comparison of the sequences of the two mutants and the WT to the reference genome results in the identification of a total number of 93,585 SNPs with appropriate quality. This is of the same order of magnitude as the results obtained by Lin *et al*. [53] (100,737 SNPs when comparing WT CC124 to the reference). We compared our data using the provided SNP library (http://stormo.wustl.edu/SNPlibrary/) and detected 18,123 unique SNPs out of 93,585 total SNPs (19%). Lin *et al*. furthermore noticed that the distribution of SNPs is not uniform across the genome, but concentrated on five chromosomes (3, 6, 12, 16 and 17), which is also in accordance to our data. These regions with high diversity seem to be a strain specific characteristic of CC124, as for example Jang and Ehrenreich [54] observed average levels of nucleotide diversity among the chromosomes in 12 natural isolates of *Chlamydomonas reinhardtii* and the two laboratory strains CC125 and CC503 (with chromosome 15 being the only exception by showing a reduction in sequence variation, probably due to large amounts of intergenic regions). However, the reason for the high accumulation of changes in CC124 on the five chromosomes remains unclear. Interestingly, most of the identified unique SNPs were located in these regions with high diversity (data not shown).

When comparing our mutants to the parental WT strain, we identified only four variants that unambiguously differed between the strains, even without preceding backcrossing to obtain isogenic strains prior to sequencing. This might be due to the extremely low rate of natural spontaneous mutations in *C. reinhardtii*, which is among the lowest rates recorded for all eukaryotes [42,55]. Furthermore the UV-mutagenesis had no observable effect on the survival of the cells. On control plates without any selection step after the UV-mutagenesis, we observed normal cell growth on agar plates and we could not detect an increase in nucleotide variations in the sequenced mutagenized strain. Indeed, high light selection was carried out directly after the UV-light treatment without any dark incubation and therefore, we did not prevent the light-driven DNA repair mechanism (reviewed by [56,57]).

Taken together our data show that the successful identification of the causative mutation by direct comparison of two mutants and the parental WT strain was possible due to different factors. To generate the high light tolerant mutants, no strong mutagen (or no mutagen at all) was used because in this case the phenotype could be selected for. The amount of induced mutations was therefore very low, and this effect was further enhanced by the very low natural rate of spontaneous mutations in *C. reinhardtii*. Even though we detected many different variants between our strains and the reference sequence, subtraction of strain specific background variants was sufficient to remove almost all non causative variants. The fact that two independently isolated high light tolerant mutants with the same phenotype show lesions in the same gene enabled us to identify the causative mutation. This finding was further confirmed by the analysis of backcrossed progeny, showing that the identified SNP co-segregates with the high light tolerant phenotype.

### Identification of *LRS1* reveals new perspectives for the characterization of regulatory mechanisms for high light adaptation in microalgae

Our mutants *hit1* and *hit2* both show a very interesting high light resistant phenotype. Light intensities of 2500 μmol m^-2^ s^-1^ as used in this approach, normally cause a very much retarded phototrophic growth in WT *C. reinhardtii*. Both mutants, however, did not only survive these severe conditions, but also retained most of their photosynthetic activity. From the physiological data it could be concluded that this robust phenotype seems to be connected to an improved ability for efficient non-photochemical quenching and a lower sensitivity towards certain ROS, especially singlet oxygen. It should be noted here that preliminary experiments indicated that the expression of the LHCSR3 gene, known to be playing a crucial role in NPQ in *C. reinhardtii* [58-61], is increased in the mutants in control light when compared to the wild type. In order to identify the internal factors responsible for increased NPQ activity and ROS tolerance in the *hit* mutants, further experiments are required in the future including qualitative and quantitative carotenoid analysis, estimation of intracellular ROS concentration, and different non-photochemical quenching parameters.

The identification of the gene *LRS1* as a putative factor involved in HL tolerance of *C. reinhardtii* demonstrates that the applied and described method is feasible to provide insights into genetic elements involved in certain phenotypes of interest. In accordance to our finding that *LRS1* is involved in the response to light, the expression of this gene (Cre02.g085050) was also found to be upregulated in *C. reinhardtii* after a shift from dark to light [45]. From preliminary alignments and comparisons, the LRS1 protein shows similarities to COP1, a key regulatory element in light response and signaling in plants with a comparable arrangement of a N-terminal RING domain an a C-terminal WD40 domain [46,47,62]. WD40 domains are reported to have different functions in various cellular processes such as signal transduction, RNA processing, vesicle transport, the assembly of the cytoskeleton, cell cycle mechanisms and apoptosis [43,63,64]. The second annotated functional domain is a RING domain that has been described as participating on different regulatory processes such as ubiquitination of proteins [65-68]. COP1 is a negative regulator controlling the degradation of transcription factors activated through direct interaction with photoreceptors [47,69,70]. It has been demonstrated that mutations in the WD40 domain of COP1 change the functional interaction with specific substrates leading to an enhanced or reduced activity [71,72]. Consequently it can be postulated that LRS1 could function as a regulatory light response signaling protein in *C. reinhardtii* and that the observed mutations in the WD40 domain of LRS1 influence the activity of the protein.

For the first time, a key genetic factor for HL tolerance, a parameter very important for outdoor cultivation of microalgae [73,74], has been identified in a non-GMO *C. reinhardtii* mutant strain by whole genome sequencing. These results highlight the power of the rapid, next-generation sequencing based identification method presented here. From our results no conclusion can be drawn if the single mutations in both mutants cause gain or loss of function of LRS1 as a regulatory element in high light adaptation. Future detailed biochemical and physiological analyses will be needed to deeply characterize the function of LRS1 in *C. reinhardtii*.

## Conclusion

The identification of novel mutations in untagged mutants deriving from forward genetic approaches has been greatly facilitated with the advent of next generation sequencing technologies. Nevertheless, for most recent advanced approaches, strains must be amenable to crossing and a polymorphic strain or closely related wild type is required. In addition, some phenotypes are sensitive to the genetic background and pooling of several mutants may not always be feasible.

In this work we present the identification of mutations causing a high light tolerant phenotype for the model microalga *Chlamydomonas reinhardtii*, avoiding any preceding crossing step, meiotic mapping or pooled progeny. We mutagenized the cells only to a low extent, followed by a strong selection. Due to the resulting low mutational load, we were able to identify the single causative mutation by whole genome sequencing of the closely related WT and mutant strains and subsequent removal of common variants. In this case whole genome sequencing of two independently isolated high light tolerant mutants resulted in the identification of point mutations within the same potentially functional motif of the same gene (see also: [26,39,75]).

The identification of LRS1 as a novel potential protein participating in adaptation reactions to high light tolerance offers new opportunities for future investigations by targeted reverse analysis to elucidate the regulatory mechanisms of microalgae under fluctuating light conditions. In addition to this important finding, our results provide a new strategy for forward genetic approaches in microalgae that avoids development of mutants by heterologous DNA insertion. This non-GMO strategy is of particular relevance for biotechnological approaches, including sustainable outdoor cultivation concepts.

## Methods

### *Chlamydomonas* strains and mutant generation

UV mutagenesis of *Chlamydomonas reinhardtii* WT strain (laboratory strain of CC124) was performed on a Biometra Transilluminator (312 nm, ultraviolet (UV) light). Cells were treated with UV light for 0, 3, 5, 10 or 15 minutes. From each condition, 4*10^6^ cells per dish were directly plated on HSM (High Salt Medium with 1.5% agar), provided with 2% CO_2_ in a home-built Plexiglas chamber and illuminated with 1500 μmol m^-2^ s^-1^. Single green colonies were isolated after 14 days. All strains were maintained on TAP plates (TRIS-Acetate-Phosphate medium with 1.5% agar) in a climate chamber at 40 μmol m^-2^ s^-1^ at 19°C and transferred onto fresh medium every 6 to 8 weeks. For the high light screening, cells were pre-cultured to mid-logarithmic growth in TAP medium at 80 to 100 μmol m^-2^ s^-1^ at 27–29°C, transferred to HSM and cultivated photoautotrophically under HL conditions (2500 to 3000 μmol m^-2^ s^-1^ bubbled with air and 3% CO_2_ at 27–29°C). Culture growth was determined by the optical density at 750 nm and by cell counting.

### Mutant phenotype characterization

For high light experiments, pre-cultured cells were transferred to HSM and cultivated photoautotrophically for 48 h under control light (600 μmol m^-2^ s^-1^) and diluted cultures were then cultivated under HL conditions (2500 μmol m^-2^ s^-1^). Culture growth was determined by the optical density at 750 nm and by cell counting. Chlorophyll contents were determined spectroscopically after extraction with 80% acetone according to [76].

*In vivo* oxygen evolution activity measurements were performed according to [77] at control light of 400 μmol m^-2^ s^-1^. To determine the oxygen evolution rate in high light, cells were illuminated with 2500 μmol m^-2^ s^-1^ during the measurement and the photosynthetic oxygen evolution was measured in 1 minute intervals over a period of 8 minutes. The respiration rate in the dark was measured directly before and after the high light treatment. To determine the photosynthetic yield and NPQ, cells were cultivated photoautotrophically at 400 μmol m^-2^ s^-1^ for 24 h and the chlorophyll fluorescence was recorded during a 10 minutes induction curve with actinic light of 800 to 1000 μmol m^-2^ s^-1^ with a Mini PAM (Waltz) and the fluorescence parameters were calculated according to [78] as ɸ PSII = (F_m_ˈ- F_t_)/ F_m_ˈ and NPQ = (F_m_ - F_m_ˈ)/ F_m_ˈ. The PAM measurements were repeated after 1 h HL (2500 μmol m^-2^ s^-1^) treatment. Prior to the NPQ measurements, 2 ml of the culture were incubated in the dark for 20 minutes.

The sensitivity against reactive oxygen species was tested on TAP agar plates supplemented with 2 μM rose bengal (RB) to induce singlet oxygen or 0.25 μM methyl viologen (MV) to induce superoxide anion radicals. Each strain was spotted on the plates in different concentrations (10^5^ and 10^4^ cells/spot shown in Figure 3) and growth at 100 μmol m^-2^ s^-1^ was observed daily for one week.

### Whole genome sequencing and identification of unique mutations

For DNA preparation from enriched nuclei, cell pellets of 400 ml culture were resuspended in 50 ml nebulizing buffer [79] and lysed twice in a nebulizer at 80 psi and 4°C. Nuclei were isolated according to [80], followed by DNA extraction with the DNeasy plant Mini Kit (Qiagen). Libraries and cluster generation were performed using the standard Illumina protocols (TruSeq DNA Sample Prep Kit v2 and TruSeq SR Cluster Kit v5-CS-GA). Sequencing was carried out on an Illumina Genome Analyzer IIx using three lanes of the flow-cell for the WT, two lanes for each of the mutants and one lane for the PHiX control (100-bp single-end reads). The sequencing data were submitted to the NCBI Sequence Read Archive (SRA) [81] under the BioProject accession number SRP037721 (PRJNA238037) with each sequencing file under the accession numbers SRS557198 (WT), SRS558641 (*hit1*) and SRS558642 (*hit2*).

All sequences were trimmed with Trimmomatic [82] and mapped to the JGI v5 C*hlamydomonas* assembly of the reference strain CC503 (mt+) [5] using the Burrows-Wheeler Aligner (BWA) [83]. Format conversion was done with SAMtools [84], deduplication and adding of read groups was performed with PicardTools (http://picard.sourceforge.net/). Variants were identified and filtered with GATK [85] and SAMtools mpileup [84], using the Genome Quality filter with a cutoff of GQ ≥ 20. Heterozygous variants with an allele frequency below 0.9 were removed using the command line tool awk [86]. Visualization of the data was accomplished using the ReadXplorer double track viewer [87] and the Integrated Genomics Viewer (IGV) [88], enabling direct comparison of the different alignments of each strain (including SNPs) to the JGI reference. Repeats were detected with RepeatMasker [89] and common SNPs between each mutant and the parental (WT) were deleted from the dataset using awk.

### Backcrossing and segregation analysis

Mutants *hit1* and *hit2* were backcrossed at least twice with either CC125 (mt^+^) or CC124 (mt^−^) and 59 of the resulting progeny were tested for growth under HL conditions in 20 ml HSM.

The mutation was examined in all progeny as well as in *hit1*, *hit2*, CC124, and CC125 by Sanger sequencing of a genomic DNA fragment amplified using BIO-X-ACT™ Short DNA Polymerase (BIOLINE) with primers flanking the mutation in *hit1* (Fwd: CACCGACCCGCACCTACT and Rev: AGGGACCAGAGCTTGAGG) or (Fwd: CCCTAACACACACCCTATGC and Rev: CCTAATGCACCTGACTCACC) and *hit2* (Fwd: CCTTTCTCCAACACCATGTC and Rev: AGGGACCAGAGCTTGAGG).

### *In silico* analysis of LRS1

Protein alignments were performed using the SDSC biology workbench (http://workbench.sdsc.edu/). *Ab initio* modeling of the three-dimensional structure of LRS1 was performed by applying I-TASSER [90-92] to the amino acid sequence of LRS1 (Cre02.g085050) and further processed with the software DeepView-Swiss-PdbViewer [93]. Protein alignments were performed using the CLC sequence viewer (CLC bio, a QIAGEN Company).

### Availability of supporting data

Next generation sequencing data supporting the results of this article is available in the the NCBI Sequence Read Archive (SRA) (http://www.ncbi.nlm.nih.gov/sra) under the BioProject accession number SRP037721 (PRJNA238037) with each sequencing file under the accession numbers SRS557198 (WT), SRS558641 (*hit1*) and SRS558642 (*hit2*).

## Abbreviations

HL: High light
SNPs: Single nucleotide polymorphisms
GMO: Genetically modified organisms
WT: Wildtype
UV: Ultraviolet
*hit1* and *hit2*: *high light tolerant* mutants 1 and 2
JGI: DOE Joint Genome Institute
GATK: Genome Analysis Toolkit
BWA: Burrows-Wheeler Aligner
AF: Allele frequency
LRS: Putative Light Response Signaling protein 1

## References

[1] Wijffels RH, Kruse O, Hellingwerf KJ (2013). Potential of industrial biotechnology with cyanobacteria and eukaryotic microalgae. Curr Opin Biotechnol.

[2] Skjånes K, Rebours C, Lindblad P (2013). Potential for green microalgae to produce hydrogen, pharmaceuticals and other high value products in a combined process. Crit Rev Biotechnol.

[3] Wobbe L, Remacle C. Improving the sunlight to biomass conversion efficiency in microalgal biofactories. J Biotechnol. 2014. doi:10.1016/j.jbiotec.2014.08.021. 10.1016/j.jbiotec.2014.08.021 25160918

[4] Mussgnug JH, Klassen V, Schlüter A, Kruse O (2010). Microalgae as substrates for fermentative biogas production in a combined biorefinery concept. J Biotechnol.

[5] Merchant SS, Prochnik SE, Vallon O, Harris EH, Karpowicz SJ, Witman GB (2007). The chlamydomonas genome reveals the evolution of Key animal and plant functions. Science.

[6] Blaby IK, Blaby-Haas CE, Tourasse N, Hom EFY, Lopez D, Aksoy M, Grossman A, Umen J, Dutcher S, Porter M (2014). The Chlamydomonas genome project: a decade on. Trends Plant Sci.

[7] Snow AA, Smith VH (2012). Genetically engineered algae for biofuels: a Key role for ecologists. Bioscience.

[8] Day JG, Slocombe SP, Stanley MS (2012). Overcoming biological constraints to enable the exploitation of microalgae for biofuels. Bioresour Technol.

[9] Polle JW, Kanakagiri S-D, Melis A (2003). tla1, a DNA insertional transformant of the green alga Chlamydomonas reinhardtii with a truncated light-harvesting chlorophyll antenna size. Planta.

[10] Mitra M, Kirst H, Dewez D, Melis A (2012). Modulation of the light-harvesting chlorophyll antenna size in Chlamydomonas reinhardtii by TLA1 gene over-expression and RNA interference. Philos Trans R Soc B.

[11] Kirst H, Garcia-Cerdan JG, Zurbriggen A, Ruehle T, Melis A (2012). Truncated photosystem chlorophyll antenna size in the green microalga Chlamydomonas reinhardtii upon deletion of the TLA3-CpSRP43 gene. Plant Physiol.

[12] Bonente G, Formighieri C, Mantelli M, Catalanotti C, Giuliano G, Morosinotto T (2011). Mutagenesis and phenotypic selection as a strategy toward domestication of Chlamydomonas reinhardtii strains for improved performance in photobioreactors. Photosynth Res.

[13] Mussgnug JH, Thomas-Hall S, Rupprecht J, Foo A, Klassen V, McDowall A (2007). Engineering photosynthetic light capture: impacts on improved solar energy to biomass conversion. Plant Biotechnol J.

[14] Oey M, Ross IL, Stephens E, Steinbeck J, Wolf J, Radzun KA (2013). RNAi knock-down of LHCBM1, 2 and 3 increases photosynthetic H_2_ production efficiency of the green alga *Chlamydomonas reinhardtii*. PLoS One.

[15] Beckmann J, Lehr F, Finazzi G, Hankamer B, Posten C, Wobbe L (2009). Improvement of light to biomass conversion by de-regulation of light-harvesting protein translation in Chlamydomonas reinhardtii. J Biotechnol.

[16] Perrine Z, Negi S, Sayre RT (2012). Optimization of photosynthetic light energy utilization by microalgae. Algal Res.

[17] Carvalho AP, Silva SO, Baptista JM, Malcata FX (2011). Light requirements in microalgal photobioreactors: an overview of biophotonic aspects. Appl Microbiol Biotechnol.

[18] Niyogi KK. Photoprotection and high light responses. In: The Chlamydomonas sourcebook: organellar and metabolic processes. Edited by Stern D, vol. 2: Academic Press; 2009: 847–70.

[19] Roach T, Krieger-Liszkay A (2014). Regulation of photosynthetic electron transport and photoinhibition. Curr Protein Pept Sci.

[20] Niyogi KK (1999). Photoprotection revisited: genetic and molecular approaches. Annu Rev Plant Physiol Plant Mol Biol.

[21] Barber J, Andersson B (1992). Too much of a good thing: light can be bad for photosynthesis. Trends Biochem Sci.

[22] Long SP, Humphries S, Falkowski PG (1994). Photoinhibition of photosynthesis in nature. Annu Rev Plant Physiol Plant Mol Biol.

[23] Cirulis JT, Scott JA, Ross GM (2013). Management of oxidative stress by microalgae. Can J Physiol Pharmacol.

[24] Henke K, Bowen ME, Harris MP (2013). Perspectives for identification of mutations in the zebrafish: making use of next-generation sequencing technologies for forward genetic approaches. Methods.

[25] Nowrousian M (2013). Genomsequenzierung zur Identifikation von Mutationen. BIOspektrum.

[26] Schneeberger K, Weigel D (2011). Fast-forward genetics enabled by new sequencing technologies. Trends Plant Sci.

[27] James GV, Patel V, Nordstrom KJ, Klasen JR, Salome PA, Weigel D (2013). User guide for mapping-by-sequencing in Arabidopsis. Genome Biol.

[28] Schneeberger K. Using next-generation sequencing to isolate mutant genes from forward genetic screens. Nat Rev Genet. 2014. Advance online publication. 10.1038/nrg3745 25139187

[29] Zuryn S, Jarriault S (2013). Deep sequencing strategies for mapping and identifying mutations from genetic screens. Worm.

[30] Schneeberger K, Ossowski S, Lanz C, Juul T, Petersen AH, Nielsen KL (2009). SHOREmap: simultaneous mapping and mutation identification by deep sequencing. Nat Methods.

[31] Doitsidou M, Poole RJ, Sarin S, Bigelow H, Hobert O (2010). C. Elegans mutant identification with a One-step whole-genome-sequencing and SNP mapping strategy. PLoS One.

[32] Pomraning KR, Smith KM, Freitag M (2011). Bulk segregant analysis followed by high-throughput sequencing reveals the Neurospora cell cycle gene, ndc-1, to be allelic with the gene for ornithine decarboxylase, spe-1. Eukaryot Cell.

[33] Leshchiner I, Alexa K, Kelsey P, Adzhubei I, Austin-Tse CA, Cooney JD (2012). Mutation mapping and identification by whole-genome sequencing. Genome Res.

[34] Blumenstiel JP, Noll AC, Griffiths JA, Perera AG, Walton KN, Gilliland WD (2009). Identification of EMS-induced mutations in *Drosophila melanogaster* by whole-genome sequencing. Genetics.

[35] Zuryn S, Le Gras S, Jamet K, Jarriault S (2010). A strategy for direct mapping and identification of mutations by whole-genome sequencing. Genetics.

[36] Bowen ME, Henke K, Siegfried KR, Warman ML, Harris MP (2012). Efficient mapping and cloning of mutations in zebrafish by low-coverage whole-genome sequencing. Genetics.

[37] Miller AC, Obholzer ND, Shah AN, Megason SG, Moens CB (2013). RNA-seq-based mapping and candidate identification of mutations from forward genetic screens. Genome Res.

[38] Allen RS, Nakasugi K, Doran RL, Millar AA, Waterhouse PM (2013). Facile mutant identification via a single parental backcross method and application of whole genome sequencing based mapping pipelines. Front Plant Sci.

[39] Nordström KJV, Albani MC, James GV, Gutjahr C, Hartwig B, Turck F (2013). Mutation identification by direct comparison of whole-genome sequencing data from mutant and wild-type individuals using k-mers. Nat Biotechnol.

[40] Irvine DV, Goto DB, Vaughn MW, Nakaseko Y, McCombie WR, Yanagida M (2009). Mapping epigenetic mutations in fission yeast using whole-genome next-generation sequencing. Genome Res.

[41] Förster B, Osmond CB, Boynton JE, Gillham NW (1999). Mutants of *Chlamydomonas reinhardtii* resistant to very high light. J Photochem Photobiol B Biol.

[42] Ness RW, Morgan AD, Colegrave N, Keightley PD (2012). Estimate of the spontaneous mutation rate in *Chlamydomonas reinhardtii*. Genetics.

[43] Stirnimann CU, Petsalaki E, Russell RB, Müller CW (2010). WD40 proteins propel cellular networks. Trends Biochem Sci.

[44] Murzin AG (1992). Structural principles for the propeller assembly of ß-sheets: the preference for seven-fold symmetry. Proteins: Struct Funct Bioinf.

[45] Duanmu D, Casero D, Dent RM, Gallaher S, Yang W, Rockwell NC (2013). Retrograde bilin signaling enables *Chlamydomonas* greening and phototrophic survival. Proc Natl Acad Sci U S A.

[46] Deng XW, Matsui M, Wei N, Wagner D, Chu AM, Feldmann KA (1992). COP1, an Arabidopsis regulatory gene, encodes a protein with both a zinc-binding motif and a G beta homologous domain. Cell.

[47] Lau OS, Deng XW (2012). The photomorphogenic repressors COP1 and DET1: 20 years later. Trends Plant Sci.

[48] Galván A, González-Ballester D, Fernández E (2007). Insertional mutagenesis as a tool to study genes/functions in *Chlamydomonas*. Adv Exp Med Biol.

[49] Carlson CM, Largaespada DA (2005). Insertional mutagenesis in mice: new perspectives and tools. Nat Rev Genet.

[50] Sarin S, Prabhu S, O’Meara MM, Pe’er I, Hobert O (2008). *Caenorhabditis elegans* mutant allele identification by whole-genome sequencing. Nat Methods.

[51] Hartwig B, James GV, Konrad K, Schneeberger K, Turck F (2012). Fast isogenic mapping-by-sequencing of ethyl methanesulfonate-induced mutant bulks. Plant Physiol.

[52] Dutcher SK, Li L, Lin H, Meyer L, Giddings JTH, Kwan AL (2012). Whole-genome sequencing to identify mutants and polymorphisms in *Chlamydomonas reinhardtii*. G3 (Bethesda).

[53] Lin H, Miller ML, Granas DM, Dutcher SK (2013). Whole genome sequencing identifies a deletion in protein phosphatase 2A that affects its stability and localization in *Chlamydomonas reinhardtii*. PLoS Genet.

[54] Jang H, Ehrenreich IM (2012). Genome-wide characterization of genetic variation in the unicellular: green alga *Chlamydomonas reinhardtii*. PLoS One.

[55] Sung W, Ackerman MS, Miller SF, Doak TG, Lynch M (2012). Drift-barrier hypothesis and mutation-rate evolution. Proc Natl Acad Sci.

[56] Sinha RP, Häder DP (2002). UV-induced DNA damage and repair: a review. Photochem Photobiol Sci.

[57] Faraji S, Dreuw A (2014). Physicochemical mechanism of light-driven DNA repair by (6–4) photolyases. Annu Rev Phys Chem.

[58] Peers G, Truong TB, Ostendorf E, Busch A, Elrad D, Grossman AR (2009). An ancient light-harvesting protein is critical for the regulation of algal photosynthesis. Nature.

[59] Bonente G, Howes BD, Caffarri S, Smulevich G, Bassi R (2008). Interactions between the photosystem II subunit PsbS and xanthophylls studied in vivo and in vitro. J Biol Chem.

[60] Bonente G, Ballottari M, Truong TB, Morosinotto T, Ahn TK, Fleming GR (2011). Analysis of LhcSR3, a protein essential for feedback de-excitation in the green alga *Chlamydomonas reinhardtii*. PLoS Biol.

[61] Niyogi KK, Truong TB (2013). Evolution of flexible non-photochemical quenching mechanisms that regulate light harvesting in oxygenic photosynthesis. Curr Opin Plant Biol.

[62] Yi C, Deng XW (2005). COP1 – from plant photomorphogenesis to mammalian tumorigenesis. Trends Cell Biol.

[63] Xu C, Min J (2011). Structure and function of WD40 domain proteins. Protein Cell.

[64] Smith TF, Clemen C, Eichinger L, Rybakin V (2008). Diversity of WD-repeat proteins. The coronin family of proteins.

[65] Freemont PS, Hanson IM, Trowsdale J (1991). A novel gysteine-rich sequence motif. Cell.

[66] Borden KLB, Freemont PS (1996). The RING finger domain: a recent example of a sequence-structure family. Curr Opin Struct Biol.

[67] Borden KLB (2000). RING domains: master builders of molecular scaffolds?. J Mol Biol.

[68] Deshaies RJ, Joazeiro CAP (2009). RING domain E3 ubiquitin ligases. Annu Rev Biochem.

[69] Chen M, Chory J, Fankhauser C (2004). Light signal transduction in higher plants. Annu Rev Genet.

[70] Wang H, Ma LG, Li JM, Zhao HY, Deng XW (2001). Direct interaction of Arabidopsis cryptochromes with COP1 in light control development. Science.

[71] Holm M, Hardtke CS, Gaudet R, Deng XW (2001). Identification of a structural motif that confers specific interaction with the WD40 repeat domain of Arabidopsis COP1. EMBO J.

[72] Maier A, Schrader A, Kokkelink L, Falke C, Welter B, Iniesto E (2013). Light and the E3 ubiquitin ligase COP1/SPA control the protein stability of the MYB transcription factors PAP1 and PAP2 involved in anthocyanin accumulation in Arabidopsis. Plant J.

[73] Scott SA, Davey MP, Dennis JS, Horst I, Howe CJ, Lea-Smith DJ (2010). Biodiesel from algae: challenges and prospects. Curr Opin Biotechnol.

[74] Béchet Q, Shilton A, Guieysse B (2013). Modeling the effects of light and temperature on algae growth: state of the art and critical assessment for productivity prediction during outdoor cultivation. Biotechnol Adv.

[75] Uchida N, Sakamoto T, Kurata T, Tasaka M (2011). Identification of EMS-induced causal mutations in a non-reference *Arabidopsis thaliana* accession by whole genome sequencing. Plant Cell Physiol.

[76] Arnon DI (1949). Copper enzymes in isolated chloroplasts: polyphenoloxidase in *Beta Vulgaris*. Plant Physiol.

[77] Wykoff DD, Davies JP, Melis A, Grossman AR (1998). The regulation of photosynthetic electron transport during nutrient deprivation in Chlamydomonas reinhardtii. Plant Physiol.

[78] Maxwell K, Johnson GN (2000). Chlorophyll fluorescence—a practical guide. J Exp Bot.

[79] Atteia A, Adrait A, Brugière S, Tardif M, van Lis R, Deusch O (2009). A proteomic survey of *Chlamydomonas reinhardtii* mitochondria sheds New light on the metabolic plasticity of the organelle and on the nature of the α-proteobacterial mitochondrial ancestor. Mol Biol Evol.

[80] Winck FV, Kwasniewski M, Wienkoop S, Mueller-Roeber B (2011). An optimized method for the isolation of nuclei from *chlamydomonas reinhardtii* (chlorophyceae). J Phycol.

[81] The Sequence Read Archive (SRA) [http://www.ncbi.nlm.nih.gov/sra]. 10.1093/nar/gkq1019 PMC3013647 21062823

[82] Lohse M, Bolger AM, Nagel A, Fernie AR, Lunn JE, Stitt M (2012). RobiNA: a user-friendly, integrated software solution for RNA-Seq-based transcriptomics. Nucleic Acids Res.

[83] Li H, Durbin R (2009). Fast and accurate short read alignment with Burrows–Wheeler transform. Bioinformatics.

[84] Li H, Handsaker B, Wysoker A, Fennell T, Ruan J, Homer N (2009). The sequence Alignment/Map format and SAMtools. Bioinformatics.

[85] DePristo MA, Banks E, Poplin R, Garimella KV, Maguire JR, Hartl C (2011). A framework for variation discovery and genotyping using next-generation DNA sequencing data. Nat Genet.

[86] Aho AV, Kernighan BW, Weinberger PJ (1987). The AWK programming language: Addison-Wesley Longman Publishing Co., Inc.

[87] ReadXplorer - visualization and analysis of mapped sequences [www.readxplorer.org] 10.1093/bioinformatics/btu205 PMC4217279 24790157

[88] Thorvaldsdóttir H, Robinson JT, Mesirov JP (2013). Integrative Genomics Viewer (IGV): high-performance genomics data visualization and exploration. Brief Bioinform.

[89] RepeatMasker Open-3.0. 1996–2010 [http://www.repeatmasker.org]

[90] Roy A, Kucukural A, Zhang Y (2010). I-TASSER: a unified platform for automated protein structure and function prediction. Nat Protocols.

[91] Roy A, Yang J, Zhang Y (2012). COFACTOR: an accurate comparative algorithm for structure-based protein function annotation. Nucleic Acids Res.

[92] Zhang Y (2008). I-TASSER server for protein 3D structure prediction. BMC Bioinformatics.

[93] Guex N, Peitsch MC (1997). SWISS-MODEL and the Swiss-PdbViewer: an environment for comparative protein modeling. Electrophoresis.

[94] Phytozome [http://www.phytozome.net/]

